# Liuwei Dihuang Decoction for primary osteoporosis: A protocol for a systematic review and meta-analysis

**DOI:** 10.1097/MD.0000000000015282

**Published:** 2019-04-19

**Authors:** Yu Liu, Ping Wang, Xiuqun Shi, Huijin Li, Xionghui Zhang, Shenghu Zeng, Zhoufa Xu, Dongqing Lai, Minling Zhang

**Affiliations:** a Department of Orthopaedics, Heyuan Hospital of Chinese Medicine; b Department of Critical Care Medicine, Beijing University of Chinese Medicine Shenzhen Hospital; c Foshan Hospital of Chinese Medicine Affiliated to Guangzhou University of Chinese Medicine, Foshan, Guangdong Province, China.

**Keywords:** Liuwei Dihuang Decoction, primary osteoporosis, protocol, systematic review

## Abstract

**Background:**

Primary osteoporosis (POP) is one kind of global disease, as a serious threat to human health. Liuwei Dihuang Decoction (LWDHD) has been recommended to treat osteoporosis alone or combined with medicine in China; however, its efficacy is unclear. The object of this systematic review and meta-analysis is to evaluate the efficacy and safety of LWDHD in the management of POP.

**Methods:**

We will search The Cochrane Library, Medline, PubMed, Elsevier, Springer, Web of Science, Ovid, WHO ICTRP, CNKI, CBM, VIP, and WanFang Database from their inception to February 2019. All randomized controlled trials (RCTs) of LWDHD for the treatment of POP will be included. The language is limited to Chinese and English. The fracture incidence will be accepted as the primary outcomes. Data synthesis, subgroup analysis, sensitivity analysis will be performed by using RevMan V.5.3.5 software.

**Results:**

A high-quality synthesis of current evidence for the treatment of POP with LWDHD will be provided from efficacy and safety.

**Conclusion:**

This systematic review will generate evidence for judging whether LWDHD is an effective and safe intervention for POP or not.

**PROSPERO registration number:**

PROSPERO CRD 4201926066.

## Abstract 2

Supplemental Digital Content is available in the text

## 1 Introduction

Osteoporosis (OP) is one of the most common systemic bone disease characterized by low bone mass, microstructural damage of bone tissue, which could lead to bone fragility and prone to fracture.^[1]^ OP could be divided into 2 major categories: primary osteoporosis (POP) and secondary osteoporosis.^[2]^ POP includes postmenopausal osteoporosis, senile osteoporosis, and idiopathic osteoporosis.^[3]^ With the aging of the social population, the incidence of POP has increased year by year. POP is a major global public health problem affecting nearly 200 million patients worldwide.^[4]^ In China, the prevalence of POP in people over 60 years old is 22.6%, and 50.0% in people over 80 years old.^[5,6]^ POP is one of the main causes of high incidence of fractures in the vertebral body, proximal, hip, pelvis and other parts of the elderly.^[7]^ The prevalence of vertebral fractures in women over 50 years old in China is 15%, while it can be as high as 36.6% in women over 80 years old.^[8]^ The incidence of hip fractures in men and women over 50 years old is 129/100,000 and 229/100,000 respectively.^[9–11]^ It is estimated that the main osteoporotic fractures (wrist, vertebral body, and hip) in China were about 2.69 million in 2015, about 48,300 in 2035, and about 5.99 million in 2050.^[12]^ Osteoporotic fractures are extremely harmful, which are one of the main causes of disability and death in elderly patients. Within 1 year after hip fracture, 20% of patients will die due to various complications; about 50% of patients will have disability, which result in the reduction in the quality of life significantly. Osteoporotic fractures will cause physical and psychological damage to the patients, and will also increase the burden to the family and society.^[13,14]^ According to prediction, the medical expenses for major osteoporotic fractures (wrist, vertebral body, and hip) in China in 2015, 2035, and 2050 will be as high as 72 billion yuan, 132 billion yuan, and 163 billion yuan, respectively.^[12]^ At present, the treatment of POP mainly includes bisphosphonates, calcitonin, calcium carbonate D3 tablets, and alendronate sodium tablets.^[15]^ Due to the long term, high cost, adverse reaction, it is unable to meet the needs of comprehensive management of POP.^[16,17]^

Traditional Chinese medicine (TCM) is an important part of complementary and alternative medicine (CAM), which has been widely used to treat POP.^[18]^ Liuwei Dihuang Decoction (LWDHD) consists of 6 kinds of TCM (Rehmannia glutinosa [Gaetn.] Libosch. ex Fisch. et Mey, Cornus officinalis Sieb. et Zucc, Dioscorea opposita, Alisma plantago-aquatica Linn, Cortex Moutan, and Wolfiporia cocos). LWDHD has been used in the treatment of osteoporosis, however, the sample and quality of randomized controlled trials (RCTs) is small and there is no systematic reviews regarding its efficacy and safety of LWDHD in the management of POP.^[19–22]^ In this article, we aim to perform a systematic review to evaluate effectiveness and safety of LWDHD in the treatment of osteoporosis in order to provide reference for clinical application.

## 2 Methods

### 2.1 Inclusion criteria for study selection

Only RCTs published in Chinese or English regarding efficacy of LWDHD in the treatment of patients with OP will be included regardless of allocation concealment or blinding. Nonrandomized controlled trials, animal experiments, case reports, reviews, and abstracts will be excluded.

#### 2.1.1 Types of patients

Gender and age are not limited.

Patients diagnosed as POP according to Consensus of experts on the diagnostic criteria of osteoporosis in China (3rd draft—2014 edition) developed by the Osteoporosis Committee of the Chinese Society of Gerontology.^[23]^ There are no limitations in age, gender, ethnicity, and severity of the disease. Patients with secondary osteoporosis, severe cardiovascular disease, bone metabolic disease, Parkinson's disease, cognitive impairment, and severe mental disease will be excluded.

#### 2.1.2 Types of interventions

The experimental group must have been treated using LWDHD or combined with routine treatment recommended by guidelines, and the control group must have received placebo or routine treatment alone. If both groups have received routine treatment, the routine treatment must be consistent. There are no limitations in dosage and administration route of LWDHD. The course of treatment is at least 3 months. Any studies including other herbal Chinese medicine, proprietary Chinese medicine, acupuncture and acupoint application will be excluded.

#### 2.1.3 Types of outcome measures

##### 2.1.3.1 Primary outcomes

The incidence of fractures.

##### 2.1.3.2 Secondary outcomes

Bone mineral density (BMD), visual analog pain (VAS) score, urinary calcium creatinine ratio (U-Ca/Cr), alkaline phosphatase (ALP), calcium ion (Ca^2+^), phosphorus ion, osteocalcin (OC), cross-linked C-telopeptide of type I collagen (β-CTX), procollagen type I N-terminal propeptide (PINP), type I pro-glue C-terminal peptide (SCTX), and adverse reactions.

### 2.2 Search methods for the identification of studies

Relevant RCTs of LWDHD for POP in the databases including the Cochrane Library, Medline, PubMed, Elsevier, Springer, Web of Science, Ovid, World Health Organization International Clinical Trial Registration Platform (WHO ICTRP), China National Knowledge Infrastructure (CNKI), Chinese Biomedical Literature Database (CBM), Chinese Science and Technology Periodical database (VIP), and WanFang Database will be searched from their inception to February 2019. The search terms will be as follows: LWDHD, POP, and RCTs. English database will be searched by subject words, free words combined with keywords. The strategy for searching the PubMed will be shown as an example in Appendix A (Supplemental Appendix A), and modified by using other databases.

#### 2.2.1 Searching other resources

In the meantime, we will search for gray literatures such as references, related conference proceedings, and dissertations included in the study manually. What is more, we will contact experts in the field to obtain more information.

### 2.3 Data collection and analysis

#### 2.3.1 Selection of studies

**Figure 1:**
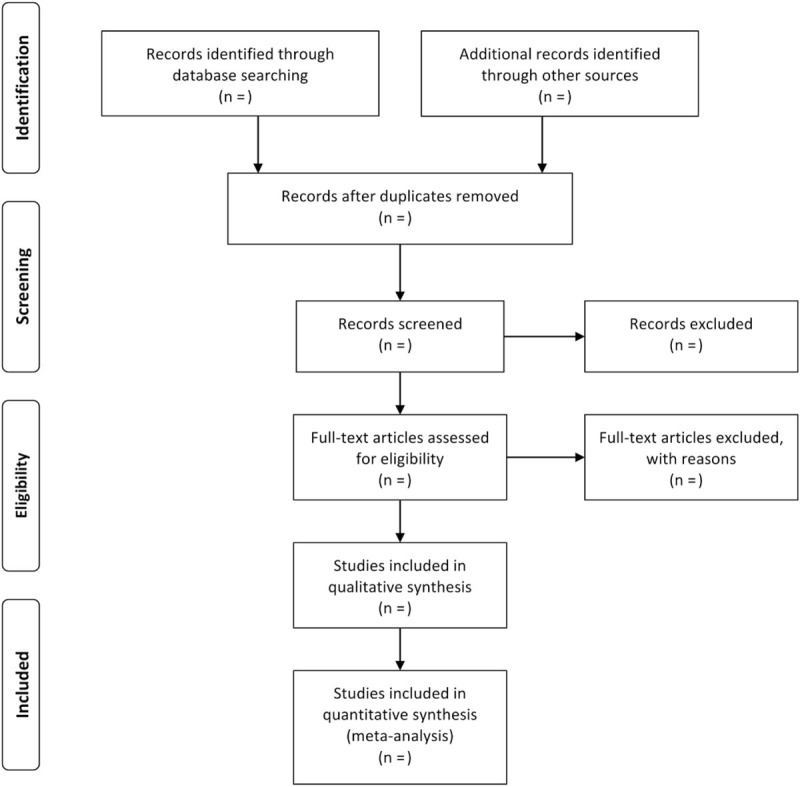
Preferred Reporting Items for Systematic review and Meta-Analysis (PRISMA) flowchart.

Two reviewers will read the titles and abstracts of all the literature independently to eliminate duplicated literature and obvious unrelated documents articles according to the inclusion criteria and exclusion criteria. Then, they will read the full text according to the inclusion and exclusion criteria to determine whether the studies would be finally included. Finally, they will doubly check the results of the included studies. When there is a disagreement, it will be solved by group discussion or consulting a third reviewer. The process of studies selection and meta-analysis is presented in an adapted PRISMA flow diagram (Fig. 1).

#### 2.3.2 Data extraction and management

Two reviewers will extract data from included literatures through prediluted extraction forms independently including includes the literature title, author, language, publication period, sample size, type of research design, basic characteristics of patients included in the study, treatment measures, intervention time, course of treatment, outcome indicators, and key factors for risk assessment of bias. Any divided opinions will be solved by group discussion or consulting a third reviewer.

#### 2.3.3 Assessment of risk of bias in included studies

Risk of bias of the included studies will be assessed according to the bias risk assessment tool recommended by Cochrane Reviewer's Handbook V.5.3 independently, including: random sequence generation, allocation concealment, blinding of participants and personnel, blinding of outcome assessment, incomplete results, selective publication, and other bias. The result will be divided into 3 levels, including low risk of bias, high risk of bias, and unclear. When there is inconsistency, problems will be resolved through discussion. If necessary, a third reviewer will be consulted.

#### 2.3.4 Measures of treatment effect

For the measurement data, the weighted mean difference (WMD) or the standardized mean difference (SMD) will be presented whereas the relative risk (RR) or odds ratio (OR) for enumeration data. A 95% confidence interval (CI) will be adopted to present the effect sizes.

#### 2.3.5 Dealing with missing data

If necessary information in the included literature is missing or unavailable, we will contact the author to obtain. If there failed, discuss and resolve, if necessary, assisted by the third researcher. If that cannot work, we will perform data synthesis on available data and discuss the possible consequence.

#### 2.3.6 Assessment of heterogeneity

Heterogeneity will be assessed by Chi-squared test (α=0.1) and with its value determined by I^2^. If *P > *.1, *I*^2^≦50%, it is considered that there is no statistical heterogeneity or the heterogeneity is small. If *P < *.1, *I*^2^ > 50%, the heterogeneity will be considered significant, further subgroup analysis or sensitivity analysis will be performed to find the source of heterogeneity.

#### 2.3.7 Assessment of reporting bias

If there are more than 10 trials included in the study, a funnel plot will be used to judge whether there is a publication bias.

#### 2.3.8 Data synthesis

Meta-analysis will be performed using RevMan 5.3 software (Version 5.3, Copenhagen: The Nordic Cochrane Center, The Cochrane Collaboration, 2014). If there is no statistic heterogeneity or heterogeneity is small, the fixed effects model will be used for analysis. If there is significant heterogeneity between the studies, subgroup analysis will be performed to find the source of heterogeneity. The random effects model will be employed for analysis when the significant clinical heterogeneity is excluded. If obvious clinical heterogeneity is observed, subgroup analysis or sensitivity analysis will be performed or only descriptive analysis.

#### 2.3.9 Subgroup analysis

Subgroup analysis according to the patients^,^ age, sex, participants, dosage, outcome measures, and treatment period will be performed to find the source of heterogeneity when significant clinical heterogeneity is observed.

#### 2.3.10 Sensitivity analysis

Sensitivity analysis will be adopted to determine the robustness of the results by ruling out studies of low quality and small sample size.

#### 2.3.11 Grading the quality of evidence

The quality of evidence for all outcomes will be evaluated using the Grading of Recommendations Assessment, Development and Evaluation (GRADE) software (Version 3.6, The GRADE Working Group, 2010).

## 3 Discussion

POP, with a large aging population, is one of the most common bone metabolic diseases affecting human health globally. Fracture is the most important complication of POP, which not only decrease the quality of life of patients seriously, but also increases the medical burden. Therefore, the effective drug should be searched to treat POP. LWDHD has been recommended to treat POP alone or combined with medicine in China, which may improve bone mineral density and inhibit bone resorption. However, the sample size of RCTs is small and the efficacy and safety are uncertain. What is more, there is no systematic reviews regarding its efficacy and safety. Hence, we intend to conduct a systematic review and meta-analysis to evaluate the efficacy and safety of LWDHD for POP, in order to provide evidence for the clinic, scientific researchers and health policy makers. However, there may be some potential limitations in this systematic review. First, this study only includes trails published in Chinese and English, which may lead to selection bias. Second, there may be a heterogeneity risk due to doses, age and the small sample size.

## Author contributions

**Conceptualization:** Ping Wang, Xiuqun Shi, Miling Zhang.

**Data curation:** Huijin Li, Xionghui Zhang.

**Funding acquisition:** Ping Wang.

**Investigation:** Shenghu Zeng.

**Methodology:** Xionghui Zhang, Zhoufa Xu.

**Project administration:** Yu Liu.

**Software:** Dongqing Lai, Shenghu Zeng.

**Supervision:** Yu Liu.

**Validation:** Minling Zhang, Zhoufa Xu, Dongqing Lai.

**Writing – original draft:** Yu Liu.

**Writing – review and editing:** Yu Liu.

## Supplementary Material

Supplemental Digital Content
