# Supplementary material for: Liuwei Dihuang Decoction for primary osteoporosis: A protocol for a systematic review and meta-analysis
Source: Medicine (Baltimore). 2019 Apr 19;98(16):e15282. doi: 10.1097/MD.0000000000015282 (PMC6494341; doi:10.1097/MD.0000000000015282)
Supplement: Supplemental Digital Content [file medi-98-e15282-s001.docx]

**Appendix A.**

***Search strategy used in PubMed database***

#1 primary osteoporosis OR senile osteoporosis OR Bone loss, age-related OR age-related osteoporosis OR involutional osteoporosis

#2 Liuwei Dihuang decoction OR liu wei di huang decoction OR Liuwei Dihuang tang OR Liuwei Dihuang yin OR Liuwei Dihuang Pill

#3 Randomized controlled trial OR clinical study OR Clin-ical Trial OR Controlled study OR Controlled Trial OR Random*Control* study OR random* Control* Trial

#1 AND #2 AND #3
